# Predictors of Urinary Tract Infections in Children and Antibiotic Susceptibility Pattern in the Buea Health District, South West Region, Cameroon

**DOI:** 10.1155/2020/2176569

**Published:** 2020-10-31

**Authors:** Che Pantalius Nji, Jules Clément Nguedia Assob, Jane-Francis Tatah Kihla Akoachere

**Affiliations:** ^1^Department of Microbiology and Parasitology, Faculty of Science, University of Buea, P.O. Box 63, Buea, South West Region, Cameroon; ^2^Department of Biomedical Sciences, Faculty of Health Sciences, University of Buea, P. O. Box, 63 Buea, South West Region, Cameroon

## Abstract

Urinary tract infections (UTI) are among the most common pediatric infections and if not promptly diagnosed and treated, it could cause long term complications. Worldwide and in Cameroon, little attention has been paid to this growing problem in the pediatric population. Identification of risk factors will contribute significantly to prevention. A cross-sectional case-control study was carried out in children ≤ 15 years to identify the risk factors of UTI, etiologic agents, and their antibiotic susceptibility. Samples (urine) were collected from in and outpatients with symptoms of UTI attending two health facilities in Buea. Controls were age- and sex-matched children in the community and those visiting these health facilities for unrelated reasons. Samples were analyzed by microscopy, culture, and antibiotic susceptibility of bacteria isolates tested by the disc diffusion technique. Questionnaires were administered to collect sociodemographic, clinical characteristics and data on risk factors. Odds ratios and bivariate and multivariate analyses were used to assess the relationship between predictors (symptoms and risk factors) and UTI. *P* < 0.05 was considered significant. A total of 405 participants (200 cases and 205 controls) were investigated. UTI prevalence was 12% in cases. From the UTI cases, bacteria was the major cause of infection, with *E. coli* (39.4%) predominating. Parasitic organisms, *Trichomonas vaginalis* (0.5%) and *Schistosoma spp* (0.5%), and yeast (6%) were also detected. Urinary urgency (*F* = 4.98, *P* = 0.027) and back pain (*F* = 12.37, *P* = 0.001) were associated to UTI following bivariate analysis. These parameters could be used to predict UTI in the pediatric population in the study area. Third generation cephalosporins: ceftriaxone (90.1%) and cefadroxil (85.4%) were the most effective and thus recommended for treatment.

## 1. Introduction

Urinary tract infections (UTIs) are among the most commonly encountered infections in the pediatric age group in both the community and hospital setting, representing 1.8% of all pediatric hospitalizations [1]. The incidence of emergency department visits for pediatric UTI is on the rise resulting in an alarming increase in emergency department expenses [2]. UTI constitutes a significant economic burden and healthcare utilization. Studies predict that about 8-10% of girls and 2-3% of boys will have symptomatic UTI before the age of 7, with frequency higher in males in their first three months of life and with increasing age, the frequency becomes higher in females than males [3]. In children, the prevalence of community-acquired urinary tract infection has been seen to vary with season [4].

Causative agents of UTI in the pediatric population are similar to those of the rest of the population. Microorganisms of the bacterial origin are the main cause of UTI, although viruses are increasingly recognized as a cause of lower UTI among immunocompromised individuals [5]. UTIs of the fungal origin are mostly caused by *Candida albicans* [6]. Parasitic diseases such as schistosomiasis and trichomoniasis give rise to renal and lower urinary tract diseases [7]. Most UTIs in children are monomicrobic, with *E. coli* being the most frequently isolated pathogen [8, 9]. Other members of the *Enterobacteriaceae* as well as *Pseudomonas aeruginosa* and species of *Staphylococcus* have also been isolated from UTI cases. However, underlying host factors such as patient's age and gender may influence the prevalence of the causative agents [10, 11]. Because the prevalence of uropathogens may vary with the age and geographic location, knowledge of local prevailing pathogens is necessary to guide disease management.

Several risk factors including age, gender, fever, constipation, uncircumcision, lack of toilet training, bladder instability, infrequent voiding of urine, previous antibiotic use, vesicoureteral reflux, and presence of nitrates in urine have been associated with UTIs in the pediatric population [8, 12–14]. In addition to these factors, studies in developing countries have also reported poor hygiene, thread worm infections, and immunocompromised state as other risk factors [15]. A recent study in Taiwan demonstrated a significant association between the obesity and urinary tract infection in children [16]. Knowledge of risk factors is necessary to reduce morbidity since UTI will be promptly diagnosed and managed. Because children with UTIs usually present with nonspecific signs and symptoms, the laboratory investigation is imperative as UTI may often be missed on the history and physical examination. If not appropriately diagnosed and well managed, UTIs may become chronic and result in scaring of the kidney, causing hypertension and renal failure.

Management of the infection is becoming progressively complicated due to increasing resistance to antibiotics by etiologic agents, whereas the initial spread of resistant pathogens was in hospital settings, and resistant pathogens have emerged in community-onset UTIs [17]. Knowledge of the regional antibiotic resistance is necessary to guide empiric therapy, particularly in resource poor settings where there are no facilities for culture and antibiotic susceptibility testing.

The evolving state of knowledge about pediatric UTI leaves many questions and controversies with a major concern being the risk factors associated with these infections. Little attention has been paid to this small but growing problem worldwide and in Cameroon. While mortality rates are not usually high, the economic burden is substantial [2]. Very few studies in Cameroon have addressed UTI in the pediatric population. Motse et al. [8] identified factors associated with UTI and their diagnostic performances in children under 5 in Douala, Cameroon. Akoachere et al. [17] investigated the etiologic profile and antibiotic susceptibility of community-acquired UTI in participants of all groups. The aim of the present study was to investigate the risk factors and etiologic agents of UTI and their susceptibility in the pediatric population (children ≤ 15 years) in Buea. Findings will enhance UTI prevention, diagnosis, and management in the pediatric population in the study area.

## 2. Materials and Methods

### 2.1. Study Area and Design

The study was carried out in Buea, the capital city of South West Region, Cameroon. Buea is located on the eastern slopes of Mount Cameroon. It has tropical and mountain rainforest as well as savannah vegetation. The climate is humid with extended periods of rainfall, characterized by incessant drizzle and damp fogs common during the rainy season. Buea has a fast-growing population of over 200,000 inhabitants of which about 25% of the population is made up of children aged 15 years and below.

In a cross-sectional case-control laboratory investigation with participants drawn from two health facilities (Regional Hospital and Solidarity Clinic, Buea) and the community, urine samples from children ≤ 15 years of age presenting with symptoms of UTI (cases) and sex- and age-matched children without symptoms suggestive of UTI (controls) were analyzed to determine the predictors of UTI and their the antibiotic susceptibility. Questionnaires were administered to participants or their parents/guardians to obtain information on demographic characteristics, symptoms, and risk factors.

Those with urinary abnormalities, chronic illness, or had been on antibiotic treatment 7 days prior to the sample collection were excluded. For the control group, age- and sex-matched children with no symptoms of UTI who gave assent and parent/guardian gave consent were recruited.

### 2.2. Sample Size Calculation

The sample size was calculated using the single population proportion formula of Kish [18]. A prevalence of UTI of 13.8% reported in a similar population [19] was used. The calculated minimum sample size was 182.8. We included 200 cases and 205 controls giving an overall population of 405.

### 2.3. Sample Collection

The method used for the sample collection depended on the age of the participant [20]. For younger children (1-3 years old), samples were collected by suprapubic aspiration while clean-catch midstream samples were collected from older participants. Suprapubic aspiration was done by trained medical personnel.

Older females were asked to sit on the toilet pot, wash their genitalia with clean water, separate the labia, and then pass out some amount of urine before placing the container to collect the midstream sample. Males were instructed to wash their genitalia and pass out some quantity of urine into the toilet before placing the container to collect the sample. Samples were collected in sterile, dry, wide-mouth, leak-proof container, clearly labelled, and then transported to the Microbiology Laboratory at the Faculty of Health Sciences, University of Buea, for analysis.

### 2.4. Questionnaire

A pretested questionnaire was administered to all participants to obtain information on demographics, symptoms, and risk factors. Information concerning risk factors like kidney infection and kidney anomaly were obtained from the participant's medical report.

### 2.5. Microscopic Examination of Samples

Ten milliliters (10 ml) of sample was centrifuged at 2000 rpm for 5 minutes and the sediments mounted on a clean slide and observed under a microscope using a ×40 objective. Samples were observed for the presence of bacteria, pathogenic parasites, and other pathological indicators like crystals and epithelial cells. The wet mount was flooded with iodine to enhance visibility especially of the ova or cysts of parasites and yeast cells [20]. Microscopy which revealed at least 5 white blood cells per high power field (HPF) and/or the presence of bacteria cells and/or yeast or parasites per HPF was suggestive of UTI [21] (Table 1).

### 2.6. Isolation and Identification of Bacteria

Samples were thoroughly mixed and cultured as described by Ibeneme et al. [21]. Sample was inoculated onto Cysteine Lactose Electrolyte Deficient (CLED) agar (Neogen Corp., Lansing USA) and incubated aerobically at 37°C for 24 hours after which cultures were observed for growth. A pure growth with counts of ≥10^5^ CFU/mL for midstream samples or growth of any number of uropathogens from suprapubic samples were considered as significant. Isolated colonies from pure culture plates were subjected to identification based on colonial characters and biochemical tests. The tests carried out were Gram staining, oxidase test, catalase test, coagulase test, growth on Mannitol Salt Agar (for Gram-positive cocci), and Triple Sugar Iron Agar (Laboratories Pronadisa CONDA, S.A) for primary identification. The API 20E (Biomérieux SA, France) kit was used to confirm the identity of *Enterobacteriaceae*.

### 2.7. Antimicrobial Susceptibility Testing

The disc diffusion technique was used to study the antibiotic susceptibility of isolates according to the recommendations of the Clinical Laboratory Standards Institute (CLSI) [22]. The following antibiotics were analyzed: trimethoprim-sulphamethoxazole (1.25/23.75 *μ*g), ampicillin (10 *μ*g), ceftriazone (30 *μ*g), amoxicillin/clavulanic acid (30 *μ*g), nitrofurantoin (300 *μ*g), and cefadroxil (30 *μ*g). These are among the antibiotics recommended by the WHO [23] for the treatment of UTIs in children. Antibiotic discs were placed on inoculated Mueller-Hinton agar (Biotech. Lab. LTD, Suffulk, UK) plates and incubated for 18 h at 37°C. After the incubation period, zones of inhibition were measured to the nearest millimeters. The results were interpreted according to the Clinical and Laboratory Standards Institute [22].

### 2.8. Ethical Considerations

This study was approved by the Faculty of Health Sciences Institutional Review Board (IRB), University of Buea. An administrative clearance was obtained from the Delegation of Public Health for the South West Region and also from participating health facilities. An informed consent was obtained from parents/guardians and assent (for older children) before collection of urine specimens.

### 2.9. Statistical Analysis

Data obtained was entered into Micro Soft Excel and exported to the software package STATA 10. Means were used for continuous variables like age while proportions were used for categorical variables like sex, presence, or absence of infection. Odds ratios and bivariate and multivariate analyses were used to assess relationships between predictors (such as symptoms and risk factors) and urinary tract infections. *P* < 0.05 was considered significant.

## 3. Results

### 3.1. Characteristics of the Study Population

A total of 405 children (200 cases and 205 controls) were enrolled in this study (Table 2). The mean age of participants was 8.17 years (range 1 to 15 years). They were divided into three age groups: ≤5 years, 6–10 years, and 11–15 years. Majority of the participants were in the age group 6–10 years. For the specific symptoms, the majority of cases reported frequent urination (79%) followed by nocturia (63%). The predominant nonspecific symptoms observed in cases were fever (62%), headache (42%), vomiting (39%,) and abdominal pain (35.5%) (Table 2). The common risk factor observed among participants was incontinence (32%) while catheterization (0.5%) was the least observed. Most of these factors were more common in cases than in controls (Table 3).

### 3.2. Microscopy of Urine Samples

Of the 200 cases, bacteriuria was detected in 105 (52.5%). Of these, 22 (21%) had significant counts (≥10/HPF). Erythrocytes were detected in 29 samples of which 9 (31%) had significant erythrocyte count. Of the 129 (64.5%) samples in which epithelial cells were detected, counts were significant in 20 (15.5%) samples. Leucocytes were detected in 70 (35%) cases but significant pyuria (≥5 leukocytes/HPF) occurred in 40 (57.1%) (Table 1). Thus, these 40 (40/200, 20%) samples were presumptively diagnosed with UTI. For the control group, no sample reported significant bacteriuria or pyuria. However, 21 (10.2%) of them had <5 leukocytes/HPF.

Microscopy also revealed the presence of parasitic organisms and yeast in some samples. *Trichomonas vaginalis* (0.5%) and *Schistosoma haematobium* (0.5%) were parasitic organisms detected. Yeast cells were found in 12 (6%) samples from cases and 1 (0.5%) control sample. Of the 14 cases with parasitic infections, 12 (6%) were coinfections with bacteriuria. The prevalence of UTI in our study based on microscopy was 12% (24/200).

### 3.3. Urine Culture

Bacterial growth was found in 105 (52.5%) case samples, of which 34 (32.4%) had counts of >10^5^ CFU/ml. None of the control samples had significant bacterial growth. Significant growth was recorded in 18 (9%) males and 16 (8%) females. This difference was not significant at P = 0.385 (Table 4). Bacterial growth was significantly more in older children (*F* = 0.82, *P* = 0.048), with the most growth (35.8%) in participants 11-15 years old. Bacterial growth was significantly associated with bacteriuria results obtained by microscopy at *P* = 0.003.

**Figure 1:**
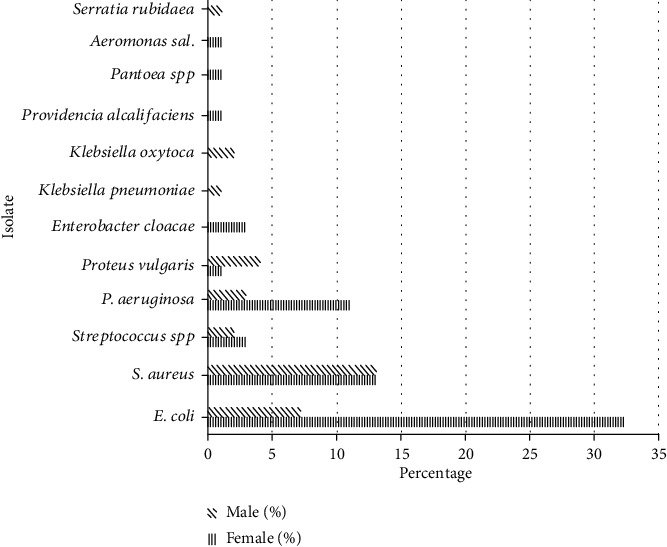
Bacteria uropathogens isolated and their distribution with respect to gender.

*Escherichia coli* (39.4%), *Staphylococcus aureus* (26.3%), and *Pseudomonas aeruginosa* (14.1%) were the most frequently isolated organisms. Other bacteria isolated were *Klebsiella oxytoca* (2.0%), *Klebsiella pneumoniae*, *Providencia alcalifaciens*, *Serratia rubidaea*, *Pantoea spp*, and *Aeromonas salmonicida* each with a frequency of isolation of 1.0% (Figure 1).

The prevalence of *E. coli* was significantly higher in females (32.3%) following bivariate (*F* = 19.02; *P* = 0.001) and multivariate (*t* = 8.63, *P* = 0.001) regression analyses. *P. aeruginosa* was higher in females (11.1%) though the difference was not significant (*F* = 0.76; *P* = 0.3846). *Serratia rubidaea, Klebsiella oxytoca*, and *Klebsiella pneumoniae* were isolated only from males while *Aeromonas salmonicida, Pantoea spp, Providencia alcalifaciens*, and *Enterobacter cloacae* were isolated only from females.

The distribution of these bacteria was found to vary with gender.

### 3.4. Predictors of UTI

Amongst the specific symptoms reported by participants, only urinary urgency (*F* = 4.98, *P* = 0.027) and back pain (*F* = 12.37, *P* = 0.001) were significantly associated with UTIs after bivariate analysis. A multivariate analysis of these symptoms also gave significant association: urinary urgency (*t* = −2.10, *P* = 0.037); back pain (*t* = 3.04, *P* = 0.003) (Table 5). None of the nonspecific symptoms showed a significant association with UTI. However, fever was reported in a majority (124, 62%) of study cases, and all 22 (11%) cases with significant bacteriuria were febrile.

Other factors investigated were age, sex, previous UTIs, frequency of occurrence ≥ twice/year, kidney infection, kidney anomaly, noncircumcision of males, catheterization, and incontinence.. Investigating the association between these factors and UTI, kidney anomaly (*P* = 0.669, OR = 3.28, CI = 0.25, 43.45) and frequency > 2/year (*P* = 0.203, OR = 2.64, CI = 0.59, 11.75) had greater odds of association with UTI amongst the test cases. However, there was no statistically significant difference from the controls (Table 6).

### 3.5. Antibiotic Sensitivity of Isolates

Overall, ceftriazone (90.1%) was the most active antibiotic, followed by cefadroxil (80.5%) and sulfamethoxazole/trimethoprim (79.3%) (Table 7). All isolates were sensitive to ceftriazone (100%) except some strains of *E. coli, S. aureus*, and *P. aeruginosa* which had resistance or intermediate activity. There was no isolate with 100% susceptibility to all drugs tested. However, some strains of *Streptococcus spp*, *Proteus vulgaris*, *Enterobacter cloacae*, *Providencia alcalifaciens*, and *Serratia rubidaea* showed 100% resistance to certain drugs. None of the predominant isolates (*E. coli* and *S. aureus*) showed 100% susceptibility to any of the drugs tested except sulfamethoxazole/trimethoprim on *S. aureus.* However, the overall susceptibility of *S. aureus* (68%) was higher than that of *E. coli* (58.1%). Ampicillin (40.5%) was the least active antibiotic (Table 7). Isolates were most resistant to nitrofurantoin (26.6%) followed by ampicillin (25.2%). With the exception of *E. coli*, all isolates had at least one drug to which they showed 100% susceptibility.

## 4. Discussion

Urinary tract infections are a common and an important clinical problem in childhood. UTI is a common cause of morbidity in children and if not diagnosed early and treated, it could result in long-term complications [24]. However, most children with UTI tend to present with fever, and this makes it difficult to distinguish UTI from other febrile illnesses on clinical grounds [25]. The goal should be to identify at-risk patients so as to put in place preventive measures against UTIs in children. Our study investigated the predictors of these UTIs in children ≤ 15 years presenting at two hospitals in Buea, the etiologic agents, and the possible antimicrobials necessary for their management.

Leukocytes were detected in 70 (35%) samples from cases of which 40 (57.1%) had significant pyuria (≥5 leukocytes/HPF) (Table 3). This indicates possible UTI. Other factors like presence of erythrocytes or bacteria cells are also considered in the case where pyuria is absent. However, the diagnosis of a UTI is primarily defined by the detection of the pathogen in urine [26]. Significant hematuria was detected in 9 (31%) samples from cases. Hematuria could be due to haemorrhage, inflammation necrosis, or trauma in the urinary system. *Schistosoma haematobium* which abrades the walls of the urinary tract causing bleeding was found in one of the samples with hematuria. Twenty (15.5%) (Table 1) case samples had epithelial cells with counts ≥ 10/HPF. It is believed that a large number of epithelial cells in urine result from inflammation of the urinary tract [20]. The nonbacterial pathogens which were detected by microscopy include *Schistosoma haematobium* (0.5%), *T. vaginalis*, (0.5) and yeast cells (6%) though their prevalence was low. *T. vaginalis* was detected in a 15-year-old female. *T. vaginalis* is a sexually transmitted infection. Apart from genital specimens, it can be detected in urine. The presence of this protozoan in our study participant could have been due to an STI rather than UTI given that some children—especially girls—become sexually active before the age of 15 years. We did not proceed to differentiate the types of yeasts. Our findings show that parasitic organisms may not be a major cause of UTI in children in the study area, compared to yeast and bacteria. It should be noted that school children or adolescents can be further exposed due to poor hygienic and sanitation in the school environment.

Only 34 out of 200 (17%) were confirmed with UTI by culture. Urine culture is definitive for diagnosis of UTIs but takes 2 to 3 days. However, microscopy is a reliable diagnostic tool in resource-poor settings such as our study area, where there are no culture facilities. There are newer technologies which could improve UTI diagnosis and management via direct pathogen detection from urine samples, rapid antimicrobial susceptibility testing, and point-of-care testing but most diagnostic laboratories cannot afford these new technologies [27].

Twenty-two out of 200 participants had bacteriuria. Non-bacterial pathogens were detected in 14 (7%) test cases of which 12 (6%) were co-infections with bacteriuria. Therefore, 24 out of 200 test cases (12%) had UTIs by microscopy, giving a UTI prevalence of 12%. Our prevalence is higher than 11% recently reported in children under 5 in Nigeria [21] but lower than 32.2% reported in a recent study of children under 5 in Douala, Cameroon [8], and 65.9% reported in Buea [17]. Though the prevalence of UTI in our study is relatively low, it is still a cause for concern given that if not promptly diagnosed and adequately treated, it could result in chronic ill health and long-term renal damage. The occurrence of these infections in children is of prime importance since they have developing kidneys which are more susceptible to be damaged by pyelonephritis.

Contrary to the report from Douala [8] and elsewhere [21, 28] in which females were more at risk than males, our study observed a higher prevalence in males than in females though the difference was not significant. Poor hygiene generally observed with male children must have accounted for this observation.

The majority of bacterial agents associated with UTIs were Gram-negative (68.6%) enteric bacteria (Figure 1). *E. coli* (39.4%) was the most frequently isolated followed by *S. aureus* (26.3%) and *P. aeruginosa* (14.1%). *E. coli* has been documented by several studies as a leading cause of bacterial UTIs in the pediatric population [8, 9, 11, 29]. These studies isolated similar uropathogens as those detected in our study. Other studies have reported *P. aeruginosa* as a major cause of UTI in pediatrics [30]. The prevalence of *E. coli* (32.3%) and *P. aeruginosa* (11.1%) was higher in females than in males (Figure 1). Similar to Saperston et al. and Naseri and Tafazoli [11, 31], gender disparity for *E. coli* was significant (df = 1, *F* = 19.02, *P* = 0.001) in pediatric patients while that for *P. aeruginosa* was negligible (df = 1, *F* = 0.76, *P* = 0.3846). The high prevalence of *E. coli* in females reported in our study contradicts the report of Ali et al. [32] who detected a higher prevalence in males than in females. Similar to the findings of Akoachere et al. [17], *P. aeruginosa* was isolated more from females than males. The second most prevalent isolate, *S. aureus*, showed an equal prevalence in females as in males. The significance of the urinary tract infection with Staphylococci in pediatric patients has been recognized by studies from other developing countries [25, 33]. *S. aureus* is an uncommon cause of pediatric UTI and has been associated with urinary malformations [34]. Although *Staphylococcus aureus* has been considered pathogenic, coagulase positive staphylococci were often interpreted as genital or skin contaminants or colonizers [31].

The clinical significance of some of the less frequently isolated bacterial pathogens in urinary tract infections such as *Aeromonas salmonicida* and *Pantoea spp* has not been very much investigated. *A. salmonicida*, though a causative agent of furunculosis, a bacterial septicaemia of salmon fish, has been isolated from a patient with UTI [35]. *Pantoea spp* has generally been associated with plants [36]; however, Cruz et al. [37] isolated one of its species, *Pantoea agglomerans*, from children with UTI.

Specific symptoms reported by participants were frequent urination, dysuria, hematuria, urgency, nocturia, incontinence, and back pain. Only urgency and back pain were significantly associated with UTI following bivariate (urgency; *F* = 4.98, *P* = 0.027, back pain; *F* = 12.37, *P* = 0.001) and multivariate (urgency; *t* = −2.10, *P* = 0.037, back pain; *t* = 3.04, *P* = 0.003) analyses (Table 5), and these could be used to predict UTI in children in our study area. No nonspecific symptom was significantly related to UTIs (*P* > 0.05) (Table 5). However, fever (62%) was the most commonly reported nonspecific symptom. A wide range of other diseases could account for fever in children [38] apart from UTI.

Over the years, the risk factors associated with UTIs have been studied amongst adults. An adequate understanding of factors in the pediatric population is necessary for prevention, prompt diagnosis, and treatment. Among the risk factors investigated, kidney anomaly (*P* = 0.669, OR = 3.28, CI = 0.25, 43.45) and frequency > 2/year (*P* = 0.203, OR = 2.64, CI = 0.59, 11.75) showed higher odds of association with UTI. However, none of these associations were of statistical significance.

For empiric treatment to be successful, knowledge of local etiologic agents and their antibiotic susceptibility remains of prime importance. Our study investigated only antibiotics recommended by the WHO [23] for treatment of UTIs in children. Sensitivity ranged from 40.5% (ampicillin) to 90.1% (ceftriazone). High resistance to ampicillin has also been reported elsewhere [39]. The cephalosporins: cefriazone (90.1%) and cefadroxil (85.4%) had the best sensitivity and could be used for treatment. Ceftriazone (CRO) was the most efficient against all the isolates except for *P. aeruginosa* which had a sensitivity of 28.6% (Table 7) contradicting the report of Baral et al. [40] who reported a high level of resistance to cephalosporins. This confirms the fact that the susceptibility of uropathogens varies with time and location. *Streptococcus spp* showed 100% resistance to cefadroxil but had 100% susceptibility to other antibiotics studied.

The synergistic effect of the combined therapy drug regimen-trimethoprim/sulphamethoxazole was high in most of the isolates; however, an increasing rate of resistance to this drug has been reported in developing countries [39]. Of the 12 bacteria species isolated, 8 had 100% susceptibility to this drug. *P. alcalifaciens* and *Pantoea spp* were completely resistant. Amoxicillin/clavulanic acid showed no activity against *E. cloacae*, *K. pneumoniae*, and *K. oxytoca*. Amoxicillin/clavulanic acid has been recommended as first-line therapy for outpatient treatment of UTI in children [41]. Although we are reporting resistance to this drug in our study, a similar study elsewhere [42] has reported high activity (100%) of this drug on uropathogens from the pediatric population. Unlike the other antibacterials, nitrofurantoin (52.7%) and ampicillin (40.5%) had the lowest overall activity. Contrary to our study, other reports have shown nitrofurantoin to have a higher sensitivity to uropathogens [40]. However, the effectiveness of nitrofurantoin in a patient is dependent on the time of administration; the longer the delay of administration from the time of infection, the less efficient the drug became [43].

## 5. Conclusion

*E. coli* was the predominant cause of UTI in our study. The symptoms, back pain and urinary urgency, were significantly associated with UTI in the study area and could be useful in predicting UTI in pediatric patients in the study area. The cephalosporines were the most active antibiotics, hence could be of value in the treatment of pediatric UTI.

This study provides baseline data which could help in the establishment of local guidelines for prediction and management of UTIs to avert complications that may arise due to UTI.

## Figures and Tables

**Table 1:** Microscopy (wet mount) of sample.

Cells	Sample group	Positive samples (%)			Statistics
		<5/HPF	≥5/HPF	Total	df, *F*, *P*
Leucocytes/pus	Test group	30 (42.9)	40 (57.1) (pyuria)	70 (35)	2, 0.78, 0.031
	Control group	21 (100)	0 (0.0)	21 (10.2)	
		< 10/HPF	≥10/HPF	Total (%)	
Bacteria cells	Test group	83 (79)	22 (21)	105 (52.5)	2, 0.81, 0.009
	Control group	34 (100)	0 (0.0)	34 (16.6)	
RBCs	Test group	20 (69)	9 (31)	29 (14.5)	2, 0.41, 0.314
	Control group	0 (0.0)	0 (0.0)	0 (0.0)	
Epithelial cells	Test group	109 (84.5)	20 (15.5)	129 (64.5)	2, 0.73, 0.048
	Control group	100 (100)	0 (0.0)	100 (48.8)	
*Schistosoma haematobium*	Test group			1 (0.5)	2, 0.344, 0.782
	Control group			0 (0.0)	
*Trichomonas vaginalis*	Test group			1 (0.5)	2, 0.208, 0.823
	Control group			0 (0.0)	
Yeast cells	Test group			12 (6.0)	2, 0.798, 0.058
	Control group			1 (0.5)	

N/B: RBCs: red blood cells; HPF: high power Field.

**Table 2:** Demographic and clinical characteristics of study participants.

Characteristics		Case group (%)	Control group (%)
Demographic			
Gender	Male	97 (48.5)	100 (48.8)
	Female	103 (51.5)	105 (51.2)
	Total	200	205

Age (years)	1–5	52 (26)	45 (22.0)
	6–10	95 (47.5)	99 (48.3)
	11–15	53 (26.5)	61 (29.8)
	Total	200	205

Clinical			
Specific symptoms	Frequent urination	158 (79)	NA
	Dysuria	49 (24.5)	NA
	Hematuria	11 (5.5)	NA
	Urgency	52 (26)	NA
	Nocturia	126 (63)	NA
	Incontinence	68 (34)	NA
	Backpain	24 (12)	NA

Nonspecific symptoms	Fever	124 (62)	NA
	Vomiting	78 (39)	NA
	Diarrhea	37 (18.5)	NA
	Abdominal pain	71 (35.5)	NA
	Flank pain	23 (11.5)	NA
	Headache	92 (46)	NA
	Nausea^∗^	14 (7)	NA
	Anorexia	13 (6.5)	NA

NA: not applicable. ^∗^Participants who reported the presence of this symptom were those >5 years old.

**Table 3:** Risk factors observed among participants.

Risk factor	No. of test cases (*n* = 200)	%	No. of control subjects (*n* = 205)	%	Bivariate analysis *P*, ORs
Previous UTIs	19	9.5	20	9.8	0.633, 0.34
Frequency > 2/year	10	5.0	7	3.4	0.434, 1.55
Kidney infection	2	1.0	2	1.0	0.742, 0.11
Kidney anomaly	4	2.0	3	1.5	0.812, 1.28
Noncircumcision	13	6.5	9	4.4	0.675, 0.88
Catheterization	1	0.5	1	0.5	0.987, 0.21
Incontinence	68	34.0	73	35.6	0.098, 0.78

**Table 4:** Prevalence of UTI in study participants following analysis of samples by culture.

Characteristic	Sample group	Total	Positive (%) ≥ 10^5^ CFU/ml	Negative (%) < 10^5^ CFU/ml
Sex				
Males	Test group	97	18 (18.6)	79 (81.4)
	Control group	100	0 (0.0)	100 (100)
Females	Test group	103	16 (15.5)	87 (84.5)
	Control group	105	0 (0.0)	105 (100)
Total	Test group	200	34 (17)	166 (83.0)
	Control group	205	0 (0.0)	205(100)
Age				
1-5	Test group	52	5(9.6)	47 (90.4)
	Control group	55	0 (0)	55 (100)
6-10	Test group	95	10 (10.5)	85 (89.5%)
	Control group	105	0 (0)	105 (100)
11-15	Test group	53	19 (35.8)	34 (64.2)
	Control group	53	0 (0)	53 (100)
Total	Test group	200	34 (17)	166 (83)
	Control group	205	0 (0)	205 (100)

Sex: df = 1, *F* = 0.76, *P* = 0.385; age: *F* = 0.82, *P* = 0.048.

**Table 5:** Relationship of clinical characteristics investigated and occurrence of UTI.

Question	Response	Count (*n*)	Frequency (%)	Bivariate analysis *F* value, *P* value	Multivariate analysis (t value, *P* value)
Specific symptoms					
Frequency	Yes	158	79	0.19, 0.661	ND
	No	42	21		
Dysuria	Yes	49	24.5	1.00, 0.319	ND
	No	151	75.5		
Hematuria	Yes	11	5.5	3.11, 0.079	ND
	No	189	94.5		
Urgency	Yes	52	26	4.98, 0.027	-2.10, 0.037
	No	148	74		
Nocturia	Yes	126	63	0.03, 0.871	ND
	No	74	37		
Incontinence	Yes	68	34	0.02, 0.880	ND
	No	132	66		
Back pain	Yes	24	12	12.37, 0.001	3.04, 0.003
	No	176	88		
Nonspecific symptoms					
Fever	Yes	124	62	0.00, 0.975	ND
	No	76	38		
Vomiting	Yes	78	39	2.45, 0.119	ND
	No	122	61		
Diarrhea	Yes	37	18.5	1.72, 0.194	ND
	No	163	81.5		
Abdominal pain	Yes	71	35.5	0.57, 0.453	ND
	No	129	64.5		
Flank pain	Yes	23	11.5	0.41, 0.522	ND
	No	177	88.5		
Headache	Yes	92	46	0.06, 0.811	ND
	No	108	54		
Nausea	Yes	14	7	0.73, 0.392	ND
	No	186	93		
Anorexia	Yes	13	6.5	0.03, 0.870	ND
	No	187	93.5		
Duration of symptoms/	≤ one week	171	85.5	0.11, 0.741	ND
	> one week and < one month	26	13		
	≥ one month	3	1.5		

**Table 6:** Association of risk factors with urinary tract infection.

Risk factors	Positive UTI cases (%)	Bivariate analysis *P*, ORs (95% CI)	Multivariate analysis (*t* value, *P* value)
*Age*			
1-5 (*n* = 52)	5 (9.6)	0.777, 0.68 (0.51, 2.41)	ND
6-10 (*n* = 95)	22 (23.2)		
11-15 (*n* = 53)	7 (13.2)		
*Sex*			
Male (*n* = 97)	18 (18.6)	0.385, 1.30 (0.33, 5.13)	ND
Female (*n* = 103)	16 (15.5)		
Previous UTIs, *n* = 19	3 (15.8)	0.311, 1.25 (0.81, 1.93)	ND
Frequency > 2/year, *n* = 10	1 (10)	0.203, 2.64 (0.59, 11.75)	ND
Kidney infection, *n* = 2	1 (50)	0.289, 0.22 (0.01, 3.61)	ND
Kidney anomaly, *n* = 4	1 (25)	0.669, 3.28 (0.25, 43.45)	ND
Noncircumcision, *n* = 13	4 (30.8)	0.191, 0.35 (0.08, 1.45)	ND
Catheterization, *n* = 1	0 (0.0)	0.421, 0.23 (0.07, 12.1)	ND
Incontinence, *n* = 68	11 (16.2)	0.880, 0.93 (0.41, 2.18)	ND

NB: NA: not applicable; ND: not determined.

**Table 7:** Susceptibility of bacteria isolates to antibiotics commonly prescribed for treatment of the urinary infection in the pediatric population.

		Cephalosporins		Nitrofurans	Folate pathway inhibitor	*β*-Lactamase inhibitor	Penicillin A	
Organism (total)	Sensitivity	CRO (%)	CFR (%)	F (%)	SXT (%)	AMC (%)	AM (%)	Overall sensitivity (%)
*E. coli*, *n* = 39	S	23 (58.9)	31 (79.5)	27 (69.2)	20 (51.3)	27 (69.2)	8 (20.5)	58.1
	I	4 (10.3)	6 (15.8)	12 (30.8)	19 (48.7)	11 (28.2)	1 (2.6)	22.7
	R	12 (30.8)	2 (4.7)	0	0	1 (2.6)	30 (76.9)	19.2
*S. aureus*, *n* = 26	S	24 (92.3)	22 (84.6)	22 (84.6)	26 (100.0)	8 (30.8)	4 (15.4)	68.0
	I	1 (3.8)	4 (15.4)	1 (3.8)	0	8 (30.8)	0	8.4
	R	1 (3.8)	0	3 (11.5)	0	10 (38.5)	22 (84.6)	23.1
*Streptococcus spp*, *n* = 5	S	5 (100.0)	0	5 (100.0)	5 (100.0)	5 (100.0)	5 (100.0)	83.3
	I	0	0	0	0	0	0	0.0
	R	0	5 (100.0)	0	0	0	0	16.7
*P. aeruginosa*, *n* = 14	S	4 (28.6)	14 (100.0)	11 (78.6)	14 (100.0)	7 (50.0)	7 (50.0)	67.9
	I	8 (57.1)	0	2 (14.3)	0	4 (28.6)	6 (42.7)	23.8
	R	2 (14.3)	0	1 (7.1)	0	3 (21.4)	1 (7.1)	8.3
*P. vulgaris*, *n* = 5	S	5 (100.0)	3 (60.0)	5 (100.0)	5 (100.0)	5 (100.0)	0	76.7
	I	0	2 (40.0)	0	0	0	0	6.7
	R	0	0	0	0	0	5 (100.0)	16.7
*E. cloacae*, *n* = 3	S	3 (100.0)	3 (100.0)	3 (100.0)	3 (100.0)	0	0	66.7
	I	0	0	0	0	0	2 (66.7)	11.1
	R	0	0	0	0	3 (100.0)	1 (33.3)	22.2
*K. pneumoniae*, *n* = 1	S	1 (100.0)	1 (100.0)	0	1 (100.0)	0	0	50.0
	I	0	0	1 (100.0)	0	1 (100.0)	1 (100.0)	50.0
	R	0	0	0	0	0	0	0.0
*K. oxytoca*, *n* = 2	S	2 (100.0)	2 (100.0)	0	2 (100.0)	0	0	50.0
	I	0	0	2 (100.0)	0	2 (100.0)	2 (100.0)	50.0
	R	0	0		0	0	0	0.0
*Providencia alcalifaciens*, *n* = 1	S	1 (100.0)	1 (100.0)	0	0	1 (100.0)	1 (100.0)	66.7
	I	0	0	0	0	0	0	0.0
	R	0	0	1 (100)	1 (100)	0	0	33.3
*Pantoea spp*, *n* = 1	S	1 (100.0)	1 (100.0)	0	0	1 (100.0)	1 (100.0)	66.7
	I	0	0	0	0	0	0	0.0
	R	0	0	1 (100.0)	1 (100.0)	0	0	33.3
*A. salmonicida*, *n* = 1	S	1 (100.0)	1 (100.0)	1 (100)	1 (100.0)	1 (100.0)	0	83.3
	I	0	0	0	0	0	1 (100.0)	16.7
	R	0	0	0	0	0	0	0.0
*Serratia rub.*, *n* = 1	S	1 (100.0)	1 (100.0)	0	1 (100.0)	1 (100.0)	1 (100.0)	83.3
	I	0	0	0	0	0	0	0.0
	R	0	0	1 (100.0)	0	0	0	16.7
Total = 99	S	71 (90.1)	80 (85.4)	74 (52.7)	78 (79.3)	56 (62.5)	28 (40.5)	
	I	13 (5.9)	12 (5.9)	18 (20.7)	19 (48.7)	26 (24.0)	13 (34.3)	
	R	15 (4.1)	7 (8.7)	7 (26.6)	2 (16.7)	17 (13.5)	61 (25.2)	

N/B: S: sensitive; I: intermediate; R: resistant; AV.: average; CRO: ceftriazone; CFR: cefadroxil; F: nitrofurantoin; SXT: sulfamethoxazole-trimethoprim; AMC: amoxicillin-clavulanic acid; AM: ampicillin.

## Data Availability

All data generated or analyzed during this study are included in this article.

## Abbreviations

UTI: Urinary tract infection
CLED: Cyseine lactose electrolyte deficient
WHO: World Health Organisation
IRB: Institutional review board
HPF: High power field
WBC: White blood cell
CRO: Ceftriazone
CFR: Cefadroxil
F: Nitrofurantoin
SXT: Sulfamethoxazole-trimethoprim
AMC: Amoxicillin-clavulanic acid
AM: Ampicillin.
